# Supplementary material for: Bovine Ultra-Long CDR H3 Specific for Bovine Rotavirus Displays Potent Virus Neutralization and Therapeutic Effects in Infected Calves
Source: Biomolecules. 2025 May 8;15(5):689. doi: 10.3390/biom15050689 (PMC12109355; doi:10.3390/biom15050689)
Supplement: Supplementary file 1 [file biomolecules-15-00689-s001.zip › biomolecules-3568281-supplementary.pdf]

Type of the Paper (Article)

Bovine Ultra-Long CDR H3 Specific for Bovine Rotavirus Displays Potent Virus Neutralization and Therapeutic Effects in Infected Calves

Qihuan Zhao [1], Puchen Li [1], Bo Wang [1], Baohui Li [1], Min Gao [1], Guanyi Ren [1], Gege Rile [1], Saqi Rila [1], Ke Ma [1] and Fuxiang Bao [1,2,*]

[1] College of Veterinary Medicine, Inner Mongolia Agricultural University, Huhhot, China
[2] Key Laboratory of Clinical Diagnosis and Treatment Techniques for Animal Disease, Ministry of Agriculture and Rural Affairs, Huhhot, China
* Correspondence: baofuxiang@imau.edu.cn

Figure S1. The serum antibody titer of immunized Bovine was measured by ELISA.

The 96-well plates were coated with BRV with a $TCID_{50}$ of 10-6.13/100 μL. Negative and blank controls (negative serum, PBS) were set up. Mouse Anti-Bovine IgG (1F2)/HRP was used as the secondary antibody to identify the serum titer of calves after the fourth immunization. As shown in Figure S1, the OD450nm value of the negative well was 0.1145 and that of the blank well was 0.0685. Therefore, based on the value of the negative well, it can be known that the antibody titer could reach 1:32000.

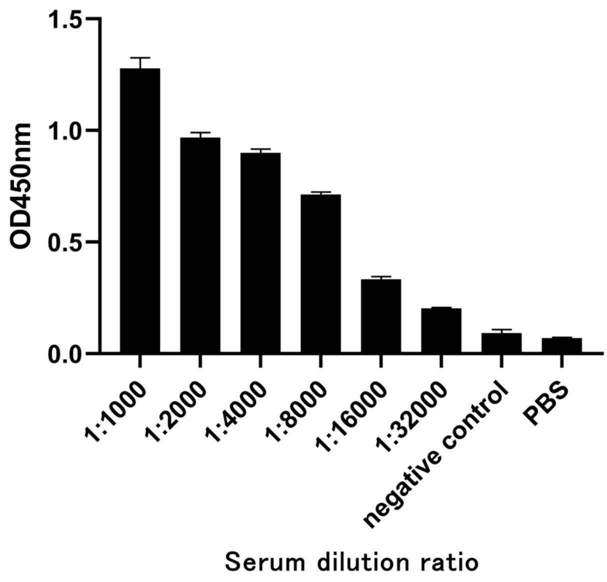

Figure S2. The enrichment of phage display library after three round of screening.

The constructed phage library was screened with BRV, and the eluted phages were obtained and then subjected to a new screening process. After three rounds of screening, phages with specific binding activity were eluted. The enrichment of BRV specific phages were calculated as input phages/output phages. The enrichment folds of the three rounds of screening were 1, 23.3, and 91.3 times respectively if compared with the first round of screening.

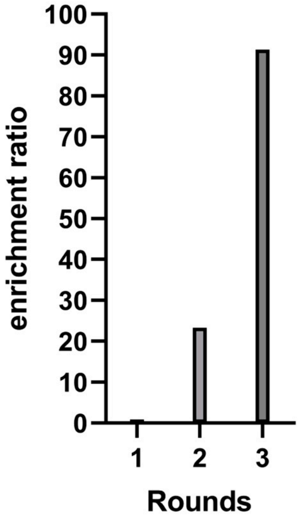

Figure S3. Colony PCR results of *pMD19-T-NSP5* recombinant plasmids.

The *BRV-NSP5* gene was ligated to pMD19-T vector, and transformed into DH5α competent cells, and single colonies were picked for PCR identification. M represent the molecular marker in the figure, and lanes 1-6 were the colonies, and lane 7 was negative control (PBS). A specific bands with the size of 127 bp were obtained from the colony PCR, and indicating that the successful construction of *pMD19-T-NSP5* recombinant plasmid.

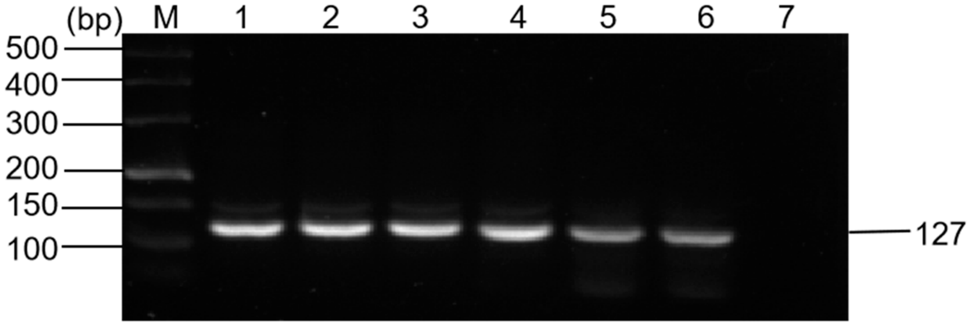

Figure S4. Survival Curve.

The mortality of calves in the calf model was analyzed, and the results are shown in the figure. The survival rates of calves in the antibody group and the PBS group were 100%. In the BRV group, calves died on the 6th and 7th days after virus challenge, and the survival rates decreased to 87.5% and 65.625% respectively.

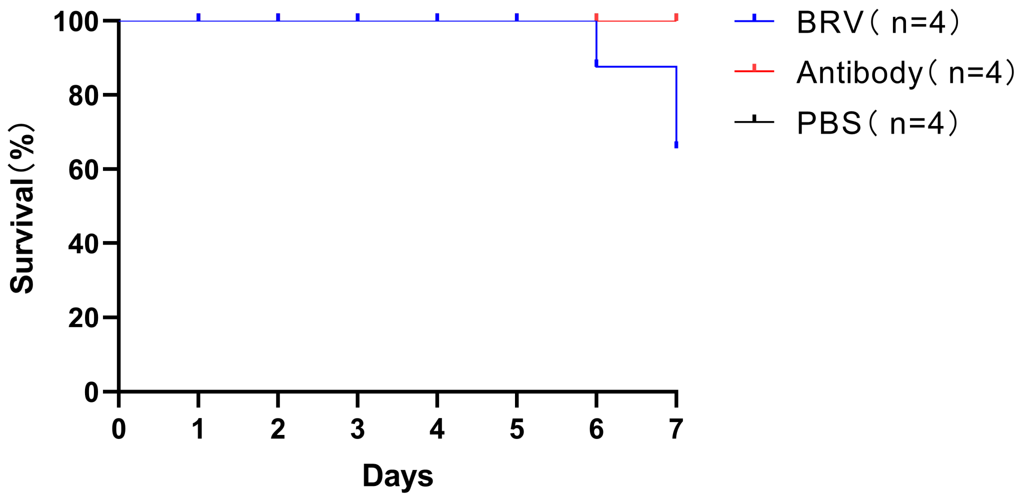

Figure S5. PCR Identification of the presence of BRV in the rectal feces of newborn calves after challenged with BRV.

PCR detection results of the antibody group, the control group and the blank group was showed in FigS5A、FigS5B and FigS5C respectively. In the figure, M represent the molecular marker, and lanes 1 - 7 represent the samples of day 1 to day 7 from the calve of each group after being BRV challenged. Lanes P and N represent the positive control (*pMD19-T-NSP5* plasmid) and the negative control (PBS) respectively. The results showed that BRV was detected in the samples of day 2 to day 4, but not in day 1 and day 5 to day 7 in the antibody group as shown in Fig. S5A. The BRV was detected in all the samples of control group from day 2 to day 7 as shown in Fig. S5B. While, BRV was not detected in all the samples of blank group as shown in Fig. S5C.

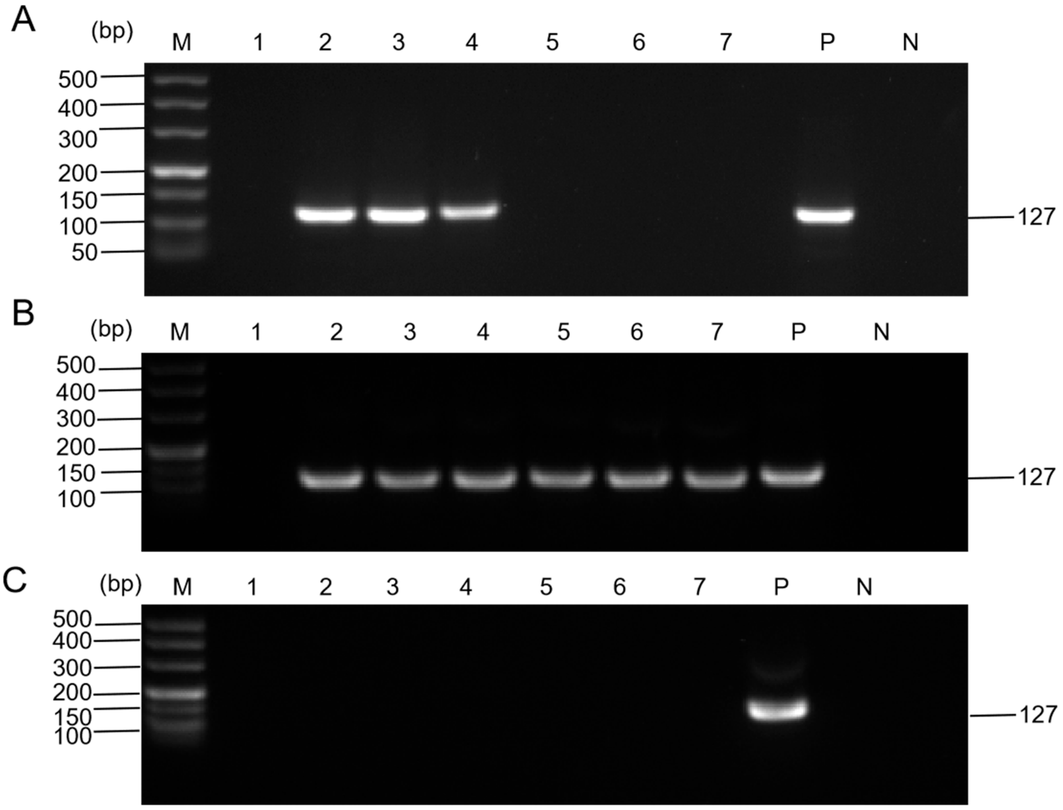

Table S1. Animal clinical symptom score table.

Rectal temperature: Fever is a primary indicator of systemic infection or inflammation. The thresholds reflect progressive severity, with higher temperatures (>40.5°C) indicating greater systemic involvement.

Mental state: Behavioral changes (e.g., lethargy, depression) correlate with worsening illness and neurological/immune responses. Stool state:

Diarrhea progression (soft → watery → bloody) reflects gastrointestinal pathology severity, with bloody diarrhea suggesting advanced enteric disease. Eating status:

Reduced appetite is a sensitive marker of morbidity; refusal to eat signals critical illness.

Table S1 shows the clinical symptom scoring table. The evaluation of clinical symptoms includes measuring rectal temperature, mental state, feeding status, and occurrences of diarrhea. Based on the symptoms described in the table, the clinical symptoms of calves can be classified into four levels.

Clinical symptoms	Asymptomatic (1)	Mild (2)	Moderate (3)	Severe (4)
**Temperature**	<39.5°C	39.6-40.0°C	40.1-40.5°C	40.6-41.5°C
**Mental State**	Normal	Lethargic	Depressed, unwilling to move	Lethargic, unresponsive to stimuli
**Stool State**	Normal	Soft, slightly loose	Watery stool	Watery stool with blood
**Eating Status**	Normal	Slightly reduced	More than half	Not eating

			reduced	

Table S2. Changes in body weight in rats.

The weight record tables of suckling rats in the BRV group and control group at 0 h, 24 h, 48 h, 84 h, 132 h and 156 h. There was no obvious weight loss or other changes in the body weight of rats in the BRV group and the control group, and body weight was increased gradually in all group of animal.

0 h

BRV Group

No. 1	10.77 g
No. 2	9.78 g
No. 3	9.98 g
No. 4	10.1 g
No. 5	10.63 g

PBS Group

No. 6	9.95 g
No. 7	10.48 g
No. 8	10.9 g
No. 9	10.28 g
No. 10	10.25 g

24 h

BRV Group

No. 1	11.92 g
No. 2	10.86 g
No. 3	11.34 g
No. 4	11.21 g
No. 5	11.56 g

PBS Group

No. 6	11.07 g
No. 7	11.58 g
No. 8	11.53 g
No. 9	11.98 g
No. 10	9.93 g

48 h

No. 1	13.08 g

No. 3	13.11 g
No. 4	13.21 g
No. 5	13.65 g

BRV Group

PBS Group

No. 6	13.08 g
No. 7	13.53 g
No. 8	13.58 g
No. 9	14.04 g

84 h

BRV Group

No. 1	19.86 g
No. 4	19.05 g
No. 5	20.15 g

PBS Group

No. 6	19.63 g
No.8	19.34 g
No. 9	19.89 g

132 h

BRV Group

No. 1	26.08 g
No. 5	25.91 g

PBS Group

No. 8	25.76g
No. 9	26.91g

156 h

BRV Group

No. 5	22.96 g

PBS Group

No. 8	23.52 g
